# Development and Proof-of-Concept Application of Genome-Enabled Selection for Pea Grain Yield under Severe Terminal Drought

**DOI:** 10.3390/ijms21072414

**Published:** 2020-03-31

**Authors:** Paolo Annicchiarico, Nelson Nazzicari, Meriem Laouar, Imane Thami-Alami, Massimo Romani, Luciano Pecetti

**Affiliations:** 1 Council for Agricultural Research and Economics (CREA), Research Centre for Animal Production and Aquaculture, viale Piacenza 29, 26900 Lodi, Italy; nelson.nazzicari@crea.gov.it (N.N.); mas.romani@libero.it (M.R.); luciano.pecetti@crea.gov.it (L.P.); 2 Ecole Nationale Supérieure Agronomique (ENSA), Laboratoire d’Amélioration Intégrative des Productions Végétales (C2711100), Rue Hassen Badi, El Harrach, Alger DZ16200, Algeria; laouar_m@yahoo.fr; 3 Institut National de la Recherche Agronomique (INRA), Centre Régional de Rabat, Av. de la Victoire, Rabat BP 415, Morocco; thamialami_ma@yahoo.fr

**Keywords:** drought tolerance, genotype × environment interaction, genetic gain, genomic selection, grain yield, inter-population predictive ability, marker-assisted selection, *Pisum sativum*

## Abstract

Terminal drought is the main stress limiting pea (*Pisum sativum* L.) grain yield in Mediterranean environments. This study aimed to investigate genotype × environment (GE) interaction patterns, define a genomic selection (GS) model for yield under severe drought based on single nucleotide polymorphism (SNP) markers from genotyping-by-sequencing, and compare GS with phenotypic selection (PS) and marker-assisted selection (MAS). Some 288 lines belonging to three connected RIL populations were evaluated in a managed-stress (MS) environment of Northern Italy, Marchouch (Morocco), and Alger (Algeria). Intra-environment, cross-environment, and cross-population predictive ability were assessed by Ridge Regression best linear unbiased prediction (rrBLUP) and Bayesian Lasso models. GE interaction was particularly large across moderate-stress and severe-stress environments. In proof-of-concept experiments performed in a MS environment, GS models constructed from MS environment and Marchouch data applied to independent material separated top-performing lines from mid- and bottom-performing ones, and produced actual yield gains similar to PS. The latter result would imply somewhat greater GS efficiency when considering same selection costs, in partial agreement with predicted efficiency results. GS, which exploited drought escape and intrinsic drought tolerance, exhibited 18% greater selection efficiency than MAS (albeit with non-significant difference between selections) and moderate to high cross-population predictive ability. GS can be cost-efficient to raise yields under severe drought.

## 1. Introduction

The combined effect of population growth, change and instability of climate, reduced available irrigation water, land degradation, and inefficient and environment-unfriendly exogenous nitrogen inputs are threatening the global food security [1,2,3]. Greater cultivation of drought-tolerant, resilient legume crops would represent a key asset for facing these challenges, by increasing the sustainability of agriculture in terms of soil fertility, energy efficiency, greenhouse gas emissions, pollution, and crop diversity on the one hand and the efficiency and quality of food systems on the other [4,5,6]. This is especially true for countries of Europe and Northern Africa, where greater legume cultivation is required also to decrease their huge dependency on international markets for high-protein feedstuff [7,8]. Plant breeding has unanimously been indicated as the main avenue to decrease the economic gap with cereal crops that limits the cultivation of grain legumes in these countries [9,10]. Drought, which has been the main abiotic stress targeted by legume improvement programmes [11], has crucial importance for most of these countries, because drought-prone environments are expected to become common throughout Southern Europe and Northern Africa and to expand northward and eastward into central Europe as a consequence of climate change [12].

Field pea (*Pisum sativum* L.) has special interest for Southern Europe, where it displays higher grain yielding ability than other rain-fed cool-season grain legumes [13]. Further assets of this crop are moderately high rates of genetic yield gain [14,15], remarkable flexibility of utilization (as grain, hay, or silage) [16], and high energy value for animal nutrition [17]. Recent work highlighted high potential interest and farmers’ appreciation for pea in North-African environments, too [16].

Genomic selection (GS) aims to predict breeding values for complex, polygenic traits by means of a statistical model constructed from phenotypic and genome-wide marker data of a germplasm sample representing a genetic base (training set), which, if sufficiently predictive, can then be applied for extensive genome-enabled selection within the target genetic base [18]. This selection strategy has represented a breakthrough for cattle production improvement [19]. The development of a high-throughput genotyping technique such as genotyping-by-sequencing (GBS) [20], by which large germplasm sets can be genotyped by thousands of single nucleotide polymorphism (SNP) markers at a lower cost than array-based techniques [21], has facilitated the application of GS in plant breeding, to select for overall crop performance or other complex traits (e.g., drought tolerance) rather than for specific traits linked to markers identified via comparative genomics or quantitative trait loci (QTL) discovery [22]. Pioneer studies for grain yield of legume crops were encouraging in this respect. The cross-environment predictive accuracy of top-performing GS models exceeded 0.45 for soybean breeding lines or landraces across sites of the USA [23,24], France [25], or China [26], and for white lupin landraces across Italian environments with contrasting climate or water availability [27]. It averaged 0.30 for three recombinant inbred line (RIL) populations of pea grown in climatically contrasting Italian environments [28], and 0.34 for various line populations of chickpea grown in Indian locations [29]. For grain yield of drought-prone pea germplasm, GS displayed an average predictive ability of 0.72 (estimated from intra-environment cross-validations) across three RIL populations grown in a managed-stress (MS) environment subjected to severe terminal drought [30], namely, the drought stress typical of Mediterranean-climate environments that implies increasing stress intensity during the reproductive stages of the crop cycle. Additionally, GS exhibited good ability to predict breeding values for other pea traits, such as phenology or individual seed weight [31]. On the whole, the available results suggested an advantage of GS over phenotypic selection in terms of predicted yield gains per unit time or unit cost, both for inbred and outbred legume crops [28,32]. However, information on actual yield progress derived from GS application would crucially contribute to verify the value of GS for legume yield improvement.

Higher yield of cool-season grain legumes under terminal drought may be achieved through different mechanisms that provide either drought escape or drought tolerance [11]. The report in [30] also included results of a genome-wide association study (GWAS), which revealed extensive co-localization of markers associated with high yield under stress and early flowering of pea. However, that study revealed also genetic variation for intrinsic drought tolerance (estimated as the yield deviation from the line value expected as a function of onset of flowering), along with putative QTL for this trait that could be exploited by marker-assisted selection (MAS). GS for intrinsic drought tolerance proved feasible too, although with lower predictive ability (averaging 0.27 across RIL populations) than GS for overall grain yield [30]. Intrinsic drought tolerance has greater practical interest than drought stress escape in inland regions of Southern Europe, where the exploitation of early flowering may be limited by greater susceptibility of autumn-sown early material to frost events [33].

The GS model for pea grain yield under severe terminal drought in [30], which was constructed from phenotyping data from one MS environment of Italy, would profit from refinement based on phenotyping data from drought-stressed agricultural environments. Yield data from MS environments can be valuable for phenotypic selection [34] and definition of GS models for drought-prone areas, because they are not subjected to the large genotype × year interaction caused by erratic rainfall that may feature in agricultural environments. However, a key prerequisite for their utilization is their ability to reproduce genotype yield responses as they occur in the target agricultural environments [35].

The main objectives of this study were (i) to improve the GS model for predicting pea grain yield under severe terminal drought that was reported in [30], by assessing the consistency of the phenotyping data used to build up that model with those recorded in two North-African agricultural environments, widening the amount of phenotyping data for GS model construction, and assessing cross-environment and cross-population (alias inter-population) predictive abilities; and (ii) to perform a proof-of-concept assessment of the value of the improved GS model and of MAS for intrinsic drought tolerance, on the basis of actual grain yields displayed under severe terminal drought by independent material that underwent GS, MAS, and phenotypic selection (PS). Additional objectives were (i) to assess the extent and pattern of genotype × environment (GE) interaction occurring across different drought-prone environments; and (ii) to verify the possible usefulness of a MS environment in Italy for yield-based PS targeted to North-African agricultural environments, as an indirect selection strategy that exploits the genetic correlation between a MS selection environment and the target agricultural sites.

## 2. Results

### 2.1. Multi-Environment Data Analysis of RIL Populations (Experiments 1, 2, and 3)

The site of Alger (Algeria) exhibited distinctly higher water availability over the crop cycle and higher crop mean yield than the Moroccan site of Marchouch (Table 1). The MS environment in Lodi (Italy), which aimed to generate severe terminal drought, was definitely more similar to Marchouch than Alger both for crop mean yield and water availability for the crop (Table 1). Yield values of top-yielding lines, i.e., those that could maximize the potential of each environment, confirmed that the MS environment and Marchouch were quite unfavorable (≤0.91 t/ha; Table 1) compared to Alger (3.33 t/ha).

**Figure 1:**
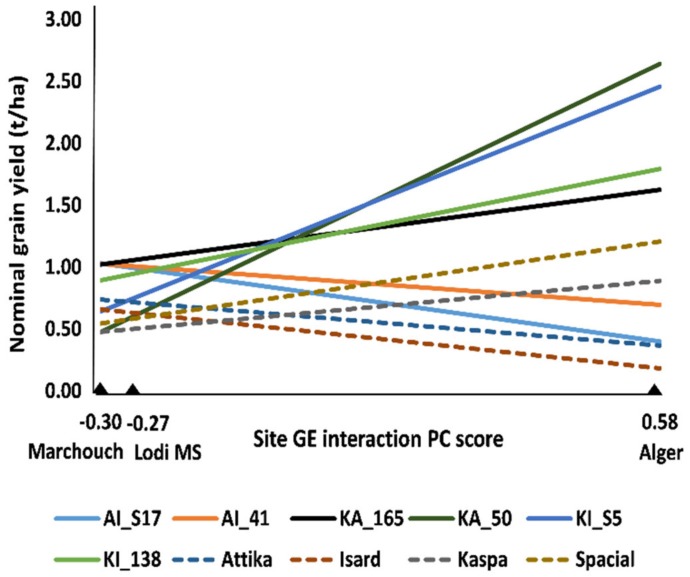
Additive Main effects and Multiplicative Interaction (AMMI)-modeled nominal grain yield of a set of top-performing pea lines out of 288 lines belonging to three connected recombinant inbred line (RIL) populations, including the two top-ranking lines in each site or over sites, three parent cultivars (Attika, Isard, and Kaspa) and one recent control cultivar (Spacial), grown in a managed drought stress (MS) environment of Lodi (Italy) and two agricultural environments of Marchouch (Morocco) and Alger (Algeria).

Among-line variation for grain yield and onset of flowering was observed within each of the three connected recombinant inbred line (RIL) populations (originated from paired crosses between the cultivars Attika, Isard, and Kaspa) in each environment (*p* < 0.05). The combined ANOVA for grain yield revealed highly significant (*p* < 0.001) genotype × environment (GE) interaction besides variation for genotype and environment main effects (Supplementary Table S1). The application of the Additive Main effects and Multiplicative Interaction (AMMI) model for partitioning the GE interaction variation showed that only the first GE interaction principal component (PC) axis was significant according to the *F_R_* test (Supplementary Table S1). AMMI-modeled line yield responses as a function of the environment score on this PC axis, which are displayed in Figure 1 for the two top-performing lines in each environment or across environments, the parent cultivars and the control cultivar Spacial, indicated (i) the remarkable yield response of some lines compared to parent or control cultivars; (ii) the large extent of GE interaction of cross-over type (i.e., implying line rank changes) across environments, and (iii) the greater similarity of the MS environment with Marchouch than with Alger for GE interaction pattern. The latter result was confirmed by the genetic correlation for yield of the whole set of lines across pairs of sites, which was moderately positive between the MS environment and Marchouch (*r_g_* = 0.50, *p* < 0.001), and non-significant (*p* > 0.05) between Alger and the MS environment (*r_g_* = −0.02) or Marchouch (*r_g_* = −0.14). Alger proved distinct from the other environments also because it displayed no phenotypic correlation of grain yield with onset of flowering (*r_p_* = −0.09, *p* > 0.10), in contrast with the negative correlation (*p* < 0.001) observed for Marchouch (*r_p_* = −0.45) and the MS environment (*r_p_* = −0.81). All these results supported the redefinition of a GS model for grain yield under severe terminal drought based on pooled phenotypic data from the MS environment and Marchouch.

Indirect PS in the MS environment targeted to Marchouch was favored by higher broad-sense heritability under MS than in the agricultural site but was hindered by the only moderate genetic correlation between the selection and the target environment (which was *r_g_* = 0.41 for values averaged across the individual RIL populations) (Table 2). As a result, indirect PS selection for Marchouch based on MS environment data was predicted to be 45% less efficient than direct PS in Marchouch (Table 2). The average *r_g_* value close to zero was responsible for the extremely low predicted efficiency of indirect PS in the MS environment relative to direct PS for Alger (Table 2).

### 2.2. Predictive Ability of Genomic Selection Models (Experiments 1, 2, and 3)

As expected, the number of available polymorphic SNP markers issued from the genotyping-by-sequencing (GBS) analysis increased as a function of the threshold of allowed genotype SNP missing data, reaching the highest value for the threshold of 50% (Data repository S1). This SNP missing data threshold implied 4364 markers for the SNP calling criterion requiring at least six aligned reads per locus, and 7521 for the criterion requiring at least four reads. Only results for the six-read criterion are reported hereafter, because this criterion provided predictive ability values that were about equal or slightly higher than those provided by the four-read criterion in all analyses. Polymorphic markers for this criterion were 165 for the SNP missing data threshold of 10%, 647 for 20%, 1713 for 30%, and 3018 for 40%.

For all yield traits, Bayesian Lasso (BL) and Ridge Regression BLUP (rrBLUP) GS models tended to display a distinct increase of predictive ability passing from 10% to 20% of genotype SNP missing data, which could be attributed to the small marker number for the 10% threshold, along with modest or nil prediction improvement beyond the 20% threshold. This is shown in Supplementary Figure S1 for models trained on pooled data of the three RIL populations.

On average, GS model training on pooled data exhibited nearly 7% higher intra-environment predictive ability than model training on the individual populations, when comparing top-performing BL or rrBLUP models for the three test environments (Table 3).

Intra-environment prediction was maximized by BL for Lodi, whereas BL and rrBLUP achieved comparable predictive ability for Alger and Marchouch (Table 3). The best-predicting GS model for line mean yield across Lodi’s MS environment and Marchouch (which was selected for GS in Exp. 4, 5, and 6) was BL trained on pooled population data with 20% SNP missing data threshold, whose predictive ability was 4% higher than that of the BL model with the same configuration but trained on individual populations (which was the alternative GS model used for Exp. 4).

In agreement with results of location similarity for GE interaction, the cross-environment predictive accuracy provided by best-predicting GS models was moderate for predicting Marchouch data from data of the MS environment or vice versa (range 0.35–0.46), and very low for predicting Alger data from MS environment data or vice versa (<0.06) or for predicting Alger data from Marchouch data or vice versa (<0.12) (Supplementary Table S2). The predictive ability of the top-performing GS model constructed from line mean yields across the MS environment and Marchouch was about 0.26 for Marchouch, 0.71 for the MS environment, and 0.03 for Alger (Table 2).

Cross-population predictive ability was investigated for the hypothesis of GS models trained on data of either one or two other connected RIL populations, considering by turns all possible combinations of training and validation populations. The assessment focused on line mean yield across Lodi’s MS environment and Marchouch (whose data were exploited for GS proof-of-concept experiment work), and line yield in Alger (whose response pattern contrasted with that observed in the other two environments). Top-performing GS models for cross-population predictive ability were generally Bayesian Lasso with 20% to 40% genotype SNP missing rate. On average, the loss of prediction for the top-performing GS model passing from intra-population prediction (Table 3) to cross-population prediction (Table 4) was only 9% (0.630 vs. 0.692) for mean yield across Lodi’s MS environment and Marchouch, and 18% (0.151 vs. 0.184) for yield in Alger, for models trained on joint data of two populations. The loss of prediction was distinctly greater, namely, 43% for mean yield across Lodi’s MS environment and Marchouch, and 46% for yield in Alger, for models trained on one population.

### 2.3. Comparison of Genomic vs. Phenotypic Selection Based on Predicted Yield Gains (Experiments 1, 2, and 3)

Compared to PS in specific environments, the predicted efficiency of GS based on the best-performing site-specific GS model was about 37% lower for Marchouch, 56% lower for Alger, and 7% higher for the MS environment (Table 2). Interestingly, the gap in predicted efficiency of GS relative to PS for Marchouch was reduced by using the GS model that incorporated also data from the MS environment besides data from Marchouch (about 31% lower efficiency; Table 2). This model reduced very slightly (2%) the advantage of GS relative to direct PS for the MS environment, while showing very low relative efficiency for Alger (Table 2).

For Marchouch, indirect selection based on the top-performing GS model was predicted to be about 24% more efficient than indirect PS based on MS environment data (as indicated by relative efficiency of 0.685 vs. 0.550; Table 2). For Alger, GS based on the site-specific model was far more efficient than indirect PS based on MS environment data (relative efficiency of 0.441 vs. 0.021; Table 2), given the inability of the MS environment to reproduce the line yield responses for this site.

### 2.4. Comparison of Genomic vs. Phenotypic Selection Based on Actual Yield Gains (Experiment 4)

This experiment aimed to compare five groups of lines selected for grain yield under severe terminal drought according to different PS or GS criteria. ANOVA results for data excluding parent lines are reported in Supplementary Table S3. The five groups of lines differed at *p* < 0.01 for grain yield and onset of flowering and at *p* < 0.05 for aerial biomass, with no line group × RIL population interaction except for onset of flowering. The selected GS model constructed from line mean yields across the MS environment and Marchouch with model training on pooled RIL data produced lines with similar grain yielding ability but somewhat lower aerial biomass and earlier flowering (*p* < 0.05) compared to the GS model trained on the individual RIL populations (Table 5).

A meaningful comparison of GS vs. PS was obtained by comparing the average grain yield progress over the mean of parent lines of the two GS procedures (0.394 t/ha) with that of PS based on line mean yields across the MS environment and Marchouch (0.399 t/ha), which implied just 1% lower yield gain of GS relative to PS (Table 5). On average, the progress over parent lines of these selections was remarkable, implying over 2.5-fold higher grain yield, associated with a distinct shift towards earlier flowering and a trend towards higher aerial biomass (Table 5). On average, GS and PS produced material with comparable aerial biomass (3.157 t/ha for GS vs. 3.216 t/ha for PS; Table 5).

Specific PS for the MS environment maximized the grain yield gain over parent lines in the same test environment (Table 5). In comparison, specific PS for Marchouch exhibited 42% lower yield progress over parent lines (0.286 vs. 0.495 t/ha; Table 5).

### 2.5. Comparison of Material with Contrasting Genomic Predictions (Experiment 5)

The three line groups evaluated in Experiment 5, which were relative to putatively top-performing, mid-performing, and bottom-performing lines according to the GS model constructed from line mean yields across the MS environment and Marchouch, differed for grain yield and onset of flowering (*p* < 0.01) but not for aerial biomass, and displayed interaction with the Cross factor for grain yield and aerial biomass (*p* < 0.05; Supplementary Table S3). On average, the lines classed by GS in the top-performing group exhibited over 2.6-fold higher grain yield and four-day earlier onset of flowering than lines classed into the mid-performing or the bottom-performing group (*p* < 0.01), with no significant difference between mid- and bottom-performing groups (Table 5). In this experiment, the yield difference between selected line and parent line groups should not be interpreted in terms of GS gain over parent lines, because the genetic base that underwent GS here was intrinsically poorly adapted to severe drought because of earlier selection for cold tolerance (unlike the RIL populations of Experiments 4 or 6). However, GS (as represented by material classed in the top-performing group) allowed for a distinct grain yield progress over the mean of parent lines (57% higher yield; Table 5) under severe drought even in this late-flowering, cold-tolerant genetic base, also by means of a remarkable shift of the selected material towards earlier onset of flowering (Table 5).

### 2.6. Comparison of Genomic Selection vs. Marker-Assisted Selection for Intrinsic Drought Tolerance (Experiment 6)

In this experiment, MAS for intrinsic drought tolerance and the GS model constructed from line mean yields across the MS environment and Marchouch were applied to a set of lines featuring similar earliness of flowering. The five line groups, which were relative to putatively top-performing, mid-performing, or bottom-performing lines according to MAS or the GS, differed for grain and aerial biomass (*p* < 0.01) and onset of flowering (*p* < 0.05; Supplementary Table S3). Both GS and MAS groups of top-performing material exhibited distinctly higher grain yield than the other groups of lines and the set of parent lines (*p* < 0.01; Table 5). Compared to top-performing material from MAS, top-performing material from GS exhibited 11% higher aerial biomass (*p* < 0.05), and 7% higher grain yield (with 18% greater selection efficiency in terms of grain yield progress over the mean of parent lines, i.e., 0.286 t/ha vs. 0.243 t/ha), but the latter difference was not significant (*p* > 0.05; Table 5).

The success of GS and MAS selections was confirmed by the progressively lower grain yield across line groups that were classed as top-performing, mid-performing, and bottom-performing, respectively (*p* < 0.05; Table 5). The fact that also GS besides MAS capitalized on genetic variation for intrinsic tolerance to drought in this experiment was confirmed by the lack of shift towards earlier onset of flowering of its material classed as top-yielding (Table 5). Indeed, the very limited variation for flowering time available for exploitation by GS in this material could justify the smaller grain yield progress over parent lines achieved by GS in this experiment relative to Experiment 4 (0.286 vs. 0.388 t/ha for GS trained on pooled data of the three RIL populations; Table 5). The difference in aerial biomass between top-performing lines issued by GS and bottom-performing lines or parent lines was greater in Experiment 6 than in Experiments 4 or 5 (Table 5), indicating that selection for intrinsic drought tolerance had a special positive impact on plant vegetative growth.

## 3. Discussion

The outstanding GE interaction of cross-over type for pea grain yield across different drought-prone environments that was highlighted by AMMI analysis and genetic correlation results represents a challenge for phenotypic or genomic selection targeting these environments. The interaction was particularly high between Alger—which could be defined as a moderate-stress environment according to the yield value around 3.3 t/ha observed for top-performing material—and the other two environments—whose yield of top-performing material was below 1 t/ha—as indicated by genetic correlations close to zero and contrasting environment ordination on GE interaction PC 1. A limitation of this study was the lack of repetition in time of the experiments in the two agricultural locations, which did not allow to assess the extent of within-site GE interaction and mean yield variation and to verify the close relationship between environment similarity for GE interaction pattern and environment mean yield that was suggested by the results. However, wide GE interaction across environment mean yields in the range of 1–3 t/ha was repeatedly observed in cool-season cereals [36,37]. In pea, GE interaction for grain yield was reportedly modest for advanced breeding lines and elite cultivars across different drought-prone environments of Australia [38] but was large for different pea material across environments of Southern Europe [28,39,40,41] as well as within a different European region such as Poland [42].

Stress escape by early flowering was a key driver of specific adaptation to severely drought-prone environments in this study, as indicated by (i) its correlation with line grain yield in the MS environment and in Marchouch and its lack of correlation with yield in Alger, and (ii) the shift towards earlier flowering of material selected by GS for yield under severe terminal drought when tested in Experiments 4 and 5. However, the concurrent importance of intrinsic drought tolerance was highlighted by results of Experiment 6, in which the distinct yield progress under severe stress that was realized by material selected via GS or MAS could hardly capitalize on stress escape by earlier flowering. In pea, intrinsic drought tolerance was reportedly associated with traits such as osmotic adjustment, greater root spread, increased stomata diffusive resistance, and proline accumulation [43,44,45]. The first two traits are also known to enhance biomass production via greater effective use of water, unlike early flowering [46], which could justify the greater increase in aerial biomass of material selected for intrinsic drought tolerance relative to that selected also for drought escape.

The moderate genetic correlation and the similarity for GE interaction pattern of the two low-yielding sites (Marchouch and the MS environment) suggested that Mediterranean-climate environments with similar drought stress extent may represent a unique target region. However, indirect PS in one environment targeted to the other environment displayed distinctly lower efficiency than direct PS. In particular, indirect selection in the MS environment targeted to Marchouch exhibited 45% lower predicted efficiency than direct PS for Marchouch, whereas material issued by PS in Marchouch displayed 42% lower actual yield gain over parent lines in the MS environment of Experiment 4 than material issued by earlier PS in the MS environment. The sizeable GE interaction across these environments, which may be due to the large difference in temperature pattern between these geographically-distant sites and the important impact that such a difference may have on GE interactions for pea grain yield [40], sets a limit to the possibility of using a MS environment in Southern Europe to select for severely stressed environments of Northern Africa. However, PS performed in a MS environment that was geographically closer to its target environments may offer advantages relative to PS in agricultural environments, because of its lower experiment error that emerged in this study and the control over year-to-year rainfall variation that it offers.

The similar performance of the tested GS models, and the negligible or nil increase of predictive ability arising from imputing population structure information, agreed with earlier results for pea [30,31]. The lack of substantial rise of predictive ability beyond 20% genotype SNP missing data (implying 647 polymorphic markers) agreed as well with earlier findings for this material [30], suggesting that moderate marker numbers may be sufficient to approach prediction maximization for biparental RIL populations because of their narrower genetic variation and slow linkage disequilibrium decay relative to a broadly-based diversity panel. Actually, one such panel exhibited high GS prediction accuracy for pea seed weight and moderate accuracy for number of seeds per plant by using only 331 well-distributed markers [31].

GS models constructed from data of severely drought-prone environments such as Marchouch and the MS environment displayed nearly no value for a moderate drought-stress site such as Alger and vice versa, indicating that GS could hardly alleviate the difficulty to cope with the large GE interaction across stress levels. Breeders could use GS (or PS) to breed for (i) specific adaptation to severe-stress or moderate-stress environments, in the presence of high rainfall variation between sites and only moderate within-site rainfall variation in their target region (as it may be the case for geographically large target regions); or (ii) wide adaptation, by selecting for average value of the breeding values predicted by one GS model for severe-stress environments and another for moderate-stress environments (or by parallel PS selection across severe-stress and moderate-stress environments), in the opposite situation. Obviously, the latter option would imply much lower genetic progress in each environment type than the former.

Cross-population predictive ability has great practical interest for breeding programmes, as the transferability of GS models for predictions in other populations would decrease the cost of model development and would impact the strategies of GS implementation. In general, cross-population predictions tend to be poor across unrelated populations of inbred crops (e.g., [47]). We envisaged two scenarios for cross-population predictions, namely, model training on one or two connected RIL populations (which imply greater potential success relative to training on RIL populations that share no common parent with the target population). Particularly for the genome-enabled prediction of yield under severe drought (object of the proof-of-concept assessment), our results indicated high transferability of models trained on two RIL populations to the third connected population, which imply substantial potential savings in model training cost when exploiting connected RIL populations. Additionally, they encourage to verify whether substantial savings of model training costs may be achieved at a modest loss of cross-population predictive ability in other situations, for example, the GS exploitation of six biparental RIL populations that originated from four parents A, B, C, and D by means of intra-population predictions for two phenotyped populations, e.g., A × B and C × D, and by cross-population predictions for the other four populations (A × C, A × D, B × C, and B × D) based on the GS model constructed from joint data of the two phenotyped populations. The moderate GS model transferability across RIL populations sharing only one parent that was indicated by the 43% loss of predictive ability relative to intra-population prediction is close to the 37% loss that was reported for grain yield across Italian agricultural environments of the same populations [28] and ensured, anyway, a moderate prediction ability (0.397; Table 4).

The results of the proof-of-concept assessment of GS based on actual yield gains in the MS environment were encouraging for genome-enabled selection. Three experiments performed on independent material indicated consistently the remarkable yield progress over parent lines of material issued by GS. Two of them, designed to compare putative top-, mid-, and bottom-performing material according to genomic estimates of breeding values (Experiments 5 and 6), confirmed the ability of GS to identify top-performing lines. Finally, Experiment 4 indicated the comparable performance in the MS environment of PS and GS for mean yield across the MS environment and Marchouch. This experiment assumed same selection intensity for GS and PS (10% selected fraction for each RIL population), which would imply greater efficiency of GS over PS when considering the lower cost per test line and the shorter selection cycle (e.g., 0.5 years vs. one year or more) of GS relative to PS. This finding agreed largely with the somewhat greater efficiency of GS over PS according to predicted yield gains, whose assessment considered the different cost per test line of these selection approaches by assuming distinct selection intensity (while not accounting for the advantage of shorter selection by GS). In an earlier study on pea, GS outperformed PS in terms of predicted efficiency per unit time (for same selection cost) and correlation with line yield responses in independent environments, for grain yield selection across agricultural environments of Northern and Central Italy subjected to GE interaction mainly due to year-to-year variation for extent of low winter temperatures [28]. A preliminary comparison in terms of actual yield gains for another legume crop, i.e., alfalfa, was less encouraging for GS, which was successful for divergent selection of higher- and lower-yielding synthetic populations but produced distinctly lower genetic gain than PS [48]. Various comparisons of GS vs. PS for crop yield were reported for cereals. For wheat yield, Lozada et al. [49] reported 32% lower actual response to selection for wheat yield from GS relative to PS, whereas Michel et al. [50] found greater prediction accuracy for independent environments of GS relative to PS. For maize yield, Beyene et al. [51] reported the greater efficiency of GS over pedigree-based conventional PS when comparing actual yield gains from GS with ordinary gains reported for PS; Beyene et al. [52] found similar actual yield gains for GS and PS in a second study; and Môro et al. [53] observed 12% greater response to selection for GS relative to PS. Additionally, Sallam and Smith [54] reported similar actual yield gains of GS and PS for barley. These cereal studies would reveal additional merit for GS once accounting for its lower cost and shorter selection cycle. A few studies [48,49] provided evidence for the advantage over PS of genomic assisted selection, by which phenotypic yield data from preliminary trials are combined with genomic predictions.

This study could define and test a GS model for severe-stress environments, while data from at least another moderate-stress site besides Alger would be needed to define a GS model for this environment type. Although preliminary, our results are not encouraging for GS targeting Alger, on the basis of the modest predictive ability and over 50% lower predicted efficiency relative to PS of the GS model constructed from one-year data.

GS did not display a distinct and statistically significant superiority over MAS for grain yield related to intrinsic drought tolerance, although its estimated selection efficiency advantage was not quite negligible when expressed in terms of yield progress over the parent lines (+18%). However, GS produced material with significantly greater aerial biomass than MAS. Earlier comparisons of GS vs. MAS for production traits were reported for non-legume crops, where GS proved more efficient but with quite variable advantage. For example, the advantage of GS was in the range 18%–43% according to simulation results [55] and 14%–50% according to actual selection responses [56] for maize, while being over 2.5-fold according to wheat selection gains [49]. The only modest disadvantage of MAS relative to GS in this study suggests that the five genomic regions that were targeted by MAS (see Supplementary Table S4) may include important drought tolerance genes, whose discovery may be the target of further research.

In conclusion, both PS and GS for pea grain yield in the Mediterranean region are challenged by large GE interaction, whose size tends to increase as a function of the difference across environments for drought stress extent and environment yield potential. A GS model defined for severe-stress environments exhibited greater efficiency than PS when accounting for its shorter selection cycle and lower evaluation costs, as well as moderate to high transferability across connected RIL populations. Further research is warranted to compare GS vs. PS and to confirm model transferability across RIL populations on the ground of actual yield gains in severely drought-prone agricultural environments, as well as to compare wide-adaptation vs. specific adaptation strategies for GS or PS as a function of the target region of a breeding programme.

## 4. Materials and Methods

### 4.1. Multi-Environment Phenotyping and Data Analysis of RIL Populations (Experiments 1, 2, and 3)

Phenotyping data were generated for 288 semi-dwarf, semi-leafless lines belonging to three connected RIL populations originated by single-seed descent from paired crosses between Attika (a European cultivar described as a spring-type), Isard (a French winter-type cultivar), and Kaspa (an Australian cultivar). These parent cultivars displayed fairly similar phenology and cycle duration along with high and stable grain yield and other positive agronomic characteristics across environments of Northern and Southern Italy [39,57]. The RIL populations are coded henceforth as ‘A × I’, ‘K × A’, and ‘K × I’ from the initials of their respective parents. The 288 lines represented a large subset of the 315 lines, 105 for each RIL population, that were phenotyped by Annicchiarico et al. [30]. In particular, this study included 96 lines for the A × I population, 92 for K × A, and 100 for K × I, for which enough seed was available for sowing in both North-African environments. In addition, the evaluation trials included the three parent cultivars, as well as the recent cultivar Spacial, which is characterized by excellent adaptation to Italian environments [41].

The lines were phenotyped for grain yield in three environments described as Exp. 1, 2, and 3 in Table 1. The first (Exp. 1) was a MS environment established in Lodi (Italy) as a large field-based phenotyping platform equipped with a rain-out shelter and a double rail irrigation boom. The management of Exp. 1, whose irrigation scheme mimicked the Mediterranean-climate rainfall pattern observed in the driest areas of Southern Italy, and its phenotyping results, were already reported in [30]. The second and third environments were the rain-fed agricultural sites of Marchouch (Exp. 2, Morocco, 33° 33′ N, 6° 41′ W) and Alger (Exp. 3, Algeria, 36° 45′ N, 3° 3′ E), respectively. Exp. 1 was sown in late winter of 2015 to avoid confounding effects of drought and cold stress, whereas Exp. 2 and 3 were autumn-sown (according to local practices) in 2015 in mild-winter environments that prevented the occurrence of cold stress. Exp. 1 involved smaller plots (0.8 × 0.2 m) and higher sowing density (100 germinating seeds/m^2^) than Exp. 2 and Exp. 3 (plot size: 1.1 × 0.8 m; sowing density: 60 germinating seeds/m^2^), owing to smaller room available in the MS environment. The experimental design was an alpha lattice with four replications for Exp. 1, and a randomized complete block (RCB) with three replications for Exp. 2 and 3. Dry grain yield was measured on a plot basis after estimating seed moisture by oven-drying seed samples at 90 °C for four days. Onset of flowering (as the number of days from April 1 to when 50% of plants in the plot had at least one open flower) was also recorded.

Grain yield and onset of flowering data of the RIL populations underwent a preliminary analysis of variance (ANOVA) that verified the occurrence of within-population variation for each experiment. Yield data of RIL material and the parent and control cultivars underwent a combined ANOVA including the factors genotype, environment, and block within environment. Experiment errors previously tested by Hartley’s test proved to be not homogeneous (*p* < 0.01), implying some loss of sensitivity for the *F* tests of genotype main effects and GE interaction in the combined ANOVA [58] that had no practical importance because of the high statistical significance (*p* < 0.001) of these effects. GE interaction variation for yield was partitioned by Additive Main effects and Multiplicative Interaction (AMMI) analysis, expressing graphically the AMMI-modeled responses as nominal yields (which exclude the site main effect, irrelevant for entry ranking) as a function of the environment score on the first GE interaction principal component (PC 1) [59]. For sake of clarity, the graph included just a subset of top-performing genotypes. The significance of GE interaction principal component (PC) axes was tested by the *F_R_* test [60]. The extent of GE interaction across pairs of environments was estimated by the genetic correlation (*r_g_*) for yield responses of the whole set of lines as described in [61].

The interest of indirect PS for yield in the Italian MS environment for each of the two North-African environments relative to direct PS for yield in each agricultural environment was assessed by comparing predicted yield gains for each PS scenario. The predicted gain in environment *j* (represented by Alger or Marchouch) from one selection cycle of direct PS is [62]:

*ΔG_Pj_* = *i_j_ H_j_^2^σ_p(j)_* (1)

where *i_j_* is the standardized selection differential, *H_j_^2^* is the broad-sense heritability on a line mean basis, and *σ_p(j)_* is the phenotypic standard deviation of the line mean values. The predicted yield gain in environment *j* from indirect selection in environment *j’* (represented by the MS environment) is [62,63]:

*ΔG_Pj/j’_* = *i_j_’H_j_H_j’_r_g(jj’)_σ_p(j)_* (2)

where *i_j_’* is the standardized selection differential in the environment *j’*, *H_j_*, and *H_j’_* are square root values of the broad-sense heritability on a line mean basis in the environments *j* and *j’*, respectively, and *r_g(jj’)_* is the genetic correlation for line yield responses across the two environments. We assumed *i_j_* = *i_j_’* for both selection scenarios and used the ratio (*H_j_ H_j’_ r_g(jj’)_*)/*H_j_^2^* to estimate the predicted efficiency of indirect PS in the MS environment relative to direct PS in each target environment. This assessment ought to be considered as preliminary, as it could not account for GE interactions within each agricultural site arising from year-to-year climatic variation (which may be large, unlike those expected in a MS environment). Relevant *r_g_* values estimated according to [61], *H^2^* values estimated by a restricted maximum likelihood method, and relative efficiency values, were assessed separately for each RIL population, reporting the values averaged across populations. *H^2^* values were also used to compute best linear unbiased prediction (BLUP) values according to [64], which were used for subsequent GS analyses. BLUP-based values of grain yield of the test material in the three cropping environments are reported in the Data repository S1 provided as supplementary material.

Statistical analyses of phenotyping data were carried out using SAS/STAT^®^ software (SAS Institute Inc, Cary, NC, USA) [65] and, for AMMI analysis, CropStat software (International Rice Research Institute, Manila, The Philippines) [66].

### 4.2. Definition of GS and MAS Procedures

DNA was extracted from bulked stipules of four F_6_ plants per genotype. Details of DNA isolation, GBS library construction, sequencing, genotype SNP calling, and SNP data filtering were reported in [30]. In brief, we adopted Elshire et al.’s [20] GBS protocol with modifications, using the *ApeK*I restriction enzyme and KAPA Taq polymerase. Raw reads (100 bp, single end read) were quality-filtered, de-multiplexed, and trimmed to 64 bp, grouping identical reads into one tag. We retained tags with 10 or more reads across all individuals for pairwise alignment aimed to find tag pairs that differed by 1 bp. The read distribution of the paired tags in each individual was used for SNP genotype calling, which, as in [30], was performed by each of two filtering criteria that removed markers with less than four or less than six aligned reads per locus, respectively. The latter, more conservative criterion aimed to minimize the risk of imperfect SNP calling arising from residual heterozygosity in the genotyped material. Markers that were monomorphic or with minor allele frequency <2.5% were removed. The data set was filtered for increasing levels of allowed genotype SNP missing values, excluding markers whose missing rate exceeded fixed thresholds of 10%, 20%, 30%, 40%, and 50%. SNP missing data were estimated using the K-Nearest neighbors imputation algorithm (K = 4) coupled with the simple matching coefficient distance function [67]. SNP marker data for the five thresholds of genotype missing data are provided in the Data repository S1.

We considered two GS models for yield prediction that stood out for predictive ability in a previous model comparison for pea grain yield limited to Lodi’s MS environment [30], i.e., Bayesian Lasso (BL; [68]) and Ridge regression BLUP (rrBLUP; [69]). While rrBLUP assumes that the effects of all loci have a common variance, BL assumes relatively few markers with large effects, allowing different markers to have different effects and variances [70]. Bayesian models assign prior densities to markers effects, thereby inducing different types of shrinkage, obtaining the solution by sampling from the resulting posterior density [68].

For each of the two SNP calling criteria, we assessed GS models trained either on pooled data of the three RIL populations or the individual populations, with five possible genotype SNP missing data thresholds (10%, 20%, 30%, 40%, 50%). BL and rrBLUP models with different combinations of data training and SNP missing data thresholds were assessed for intra-environment predictive ability, as well as for ability to predict line mean yields across MS and Marchouch environments (whose application for actual GS of drought-tolerant lines was supported by GE interaction analysis results). We also assessed (i) the cross-environment predictive ability of GS models constructed in one environment to predict breeding values of independent lines in another environment; (ii) the cross-environment predictive accuracy *r_Ac_* of the same GS models, by which the model predictive ability *r_Ab_* is readjusted as a function of the square root of the broad-sense heritability on a line mean basis *H_j_* of the predicted environment *j* by the formula *r_Ac_* = *r_Ab_*/*H_j_* [71]; and (iii) the ability of GS models constructed from line mean yields across MS and Marchouch environments to predict yield responses of independent lines in each agricultural site. Predictive ability values were measured as Pearson’s correlation between observed and predicted phenotypes using cross-validations for the single RIL populations (to avoid bias associated with different population mean value) and then averaging results across populations. Finally, we explored the cross-population predictive ability of GS by assuming model training on data of one connected RIL population or on pooled data of two connected populations for all possible combinations of training and validation populations, for two traits of practical interest represented by yield in Alger and mean yield across MS and Marchouch environments. GS regression modelling, cross-validations, and predictive ability estimation were performed using the R package GROAN [72], adopting 50 repetitions of a 10-fold stratified cross-validation scheme for each analysis.

The MAS criterion for intrinsic drought tolerance was defined on the basis of the GWAS reported in [30] for 315 lines belonging to the same RIL populations, which found 10 linked SNP markers with association score ≥2.25. Further insight on the genomic position of these markers was obtained by aligning their sequence to the pea draft genome under construction by the International Pea Genome Sequencing Project coordinated by INRA and Tayeh et al.’s [73] consensus map [74]. MAS was based on seven aligned markers which belonged to five genomic regions (markers TP78343 and TP13485, on LG 5; TP94476, on LG 1; TP6268, on LG 3; TP63677 and TP51372, around 32-33 cM of LG 7 in Tayeh et al.’s [73] map; and TP6885, around 76-77 cM of LG 7 in Tayeh et al.’s [73] map; see Supplementary Table S4). Based on our late verification of the position of these markers on Kreplak et al.’s [75] pea reference genome using the alignment tool *bwa* [76], these markers aligned on five gene coding regions of chromosomes 2, 3, 5, and 7 (Supplementary Table S4) for which we report gene names and descriptions as available in the Pulsedb website (https://www.pulsedb.org/Analysis/989011). We envisaged MAS based on two criteria, i.e., the number of favorable alleles over the seven markers, and the number of favorable alleles over the five putative QTL belonging to the five genomic regions. Both criteria, however, identified the same sets of top- and bottom-ranking independent lines.

### 4.3. Comparison of Genomic vs. Phenotypic Selection Based on Predicted Yield Gains

An estimation of the predicted yield gain from one cycle of GS in environment *j* is [77]:

*ΔG_Gj_* = *i_j_’’r_Ac_σ_a(j)_* (3)

where *i_j_’’* is the standardized selection differential used for GS, *r_Ac_* is the GS model accuracy, and *σ_a(j)_* is the standard deviation of the line breeding values. Recalling that *r_Ac_* = *r_Ab_*/*H_j_* and *σ_a(j)_* = *σ_p(j)_ H_j_*, another expression of *ΔG_Gj_* as a function of the GS model predictive ability *r_Ab_* is:

*ΔG_Gj_* = *i_j_r_Ab_σ_p(j)_* (4)

which, when compared to the predicted gain from direct PS (*ΔG_Pj_*) reported in Equation (1), indicates that the ratio of (*i_j_’’ r_Ab_*) to (*i_j_ H_j_^2^*) could be used to estimate the predicted efficiency of GS relative to direct PS in the target environment. However, a fair comparison of PS vs. GS ought to be based on the same costs. PS based on one field experiment with three replications may imply about 2.5 [78] to 3.1 [32] greater cost per evaluated line than GBS-based GS, which, considering the average value of 2.8, implies 2.8 more test lines and 2.8 smaller selected fraction for GS relative to PS when assuming same evaluation costs. Hence, we hypothesized 10% selected fraction, i.e., *i_j_* = 1.755 [60], for PS, and 3.6% selected fraction, i.e., *i_j_’’* = 2.197, for GS, and used the ratio (2.197 *r_Ab_*)/(1.755 *H_j_^2^*) to estimate the predicted efficiency of GS relative to PS. We envisaged GS for each environment based on best-performing environment-specific models or the best-performing model constructed from line mean yields across MS and Marchouch environments, computing *r_Ab_* and *H_j_^2^* values separately for each RIL population and reporting relative efficiency values averaged across populations. Particularly for agricultural sites, this comparison of selection strategies ought to be seen as preliminary, as it could not account for within-site GE interactions across cropping seasons neither for PS (where they would be accounted for in the denominator of *H_j_^2^*) nor for GS (where they would be accounted for by assessing *r_Ab_* across different test years rather than through intra-environment cross-validations). Additionally, this comparison tended to underestimate the relative value of GS, as it did not account for the further advantage of shorter selection cycle duration offered by GS relative to PS.

### 4.4. Comparison of Genomic vs. Phenotypic Selection Based on Actual Yield Gains (Experiment 4)

In this study, GS was applied to independent lines using top-performing statistical models in the earlier assessment that were constructed from line mean yields across Lodi’s MS environment and Marchouch. GS was applied using either the top-performing model trained on joint data of the three RIL populations, or the models trained on data of each separate population (RIL population-specific models). The set of 288 RILs was split into two subsets. The former set included 30 randomly chosen lines per RIL population, which acted as independent lines for GS and PS. The latter set included the remaining lines of the three populations, whose data were used for GS model definition. For each GS model, we selected for each RIL population the three lines out of 30 that were top-ranking for predicted yield. Likewise, we selected phenotypically for each RIL population the three lines out of 30 that were top-ranking for mean yield across MS and Marchouch environments. In addition, we performed environment-specific PS for Lodi’s MS environment and for Marchouch, by selecting the three top-yielding lines out of 30 separately for each environment.

The experiment included the 45 lines issued by selecting three lines for each of three RIL populations for each of the five selection criteria (PS in the MS environment, in Marchouch, and across the two environments; GS based on population-specific and on joint-population data), and the RIL parent lines, which acted as a reference to assess yield gains. The material was evaluated for dry grain yield and aerial biomass (estimated on plant material oven-dried at 90 °C for four days) and onset of flowering in Exp. 4, which was performed in the same MS environment adopted for Exp. 1. Exp. 4 was designed as an RCB experiment with four replications, using same plot size and sowing density as Exp. 1. Compared to Exp. 1, Exp. 4 involved similar available water for the crop but over six-week later sowing (Table 1), which increased somewhat the extent of drought stress exerted on the selected material.

A first ANOVA that excluded parent line data aimed to compare the five selection criteria and to assess the interaction between selection criteria and RIL populations. It included the fixed factors line group (whose variants were defined by the selection criteria), RIL population, and line within group and RIL population, along with the random factor block. A second ANOVA that included parent lines as an additional line group and that held the factors line group, line within group and block aimed to compare each group of selected lines with the parent line group by Dunnett’s multiple mean test.

### 4.5. Comparison of Material with Contrasting Genomic Predictions (Experiment 5)

The best-predicting GS model constructed from joint-population data of line mean yields across Lodi’s MS experiment and Marchouch in the earlier assessment was applied to an independent set of 90 lines that included 30 lines from each of the same three crosses (A × I, K × A, and K × I). These new lines were issued from four generations of bulk selection under local autumn-sown field conditions in Lodi (F_2_ to F_5_ generation, starting with 800 F_2_ seeds per cross), and were genotyped as F_6_ plants by GBS as described earlier. Local selection for tolerance to low winter temperatures was expected to produce a shift towards later onset of flowering in this material relative to the mean phenology of the RIL populations issued from the same crosses. GS-based predictions were exploited to select the two top-ranking lines, two mid-ranking lines (ranks 15 and 16), and the two bottom-ranking lines out of 30 lines within each cross. The 18 selected lines and the three parent lines were evaluated for grain yield, aerial biomass, and onset of flowering in Lodi’s MS environment by an RCB experiment with four replications (Exp. 5) whose management was identical to Exp. 4. The data analysis contemplated two ANOVAs as described for Exp. 4, the only difference being the presence of a Cross factor instead of a RIL population factor in the first ANOVA (as the selection was not applied to RIL material in this case).

### 4.6. Comparison of Genomic Selection vs. Marker-Assisted Selection for Intrinsic Drought Tolerance (Experiment 6)

This study assessed both GS and MAS for intrinsic drought tolerance. The GS model was constructed using the top-predicting GS model in the earlier assessment that was trained on joint data of 192 lines belonging to the RIL populations K × A and K × I evaluated in Exp. 1, and was applied to lines of the RIL population A × I (which was selected because it displayed smaller variation in onset of flowering than the other populations in earlier work [30]). To limit the impact on grain yield of line variation for earliness, we applied GS and MAS to a subset of 24 lines out of the 96 lines available for the A × I population whose onset of flowering in Exp. 1 fell in the interval *m* ± *s*, where *m* and *s* stand for mean and standard deviation values, respectively, of the phenology trait (which implied a range of 1.1 days among the 24 test lines). GS-based predictions for this subset of lines were exploited to select the three top-ranking lines, two mid-ranking lines, and three bottom-ranking lines. Likewise, MAS was used to predict three top-ranking lines that possessed all 14 favorable alleles for the seven target markers and the five relevant genomic regions, and three bottom-ranking lines that possessed no favorable alleles. The two GS-based mid-ranking lines acted as mid-ranking material also according to the MAS criterion, as they displayed six to eight favorable alleles overall. The 14 selected lines and the two parent lines were evaluated for grain yield, aerial biomass, and onset of flowering in Lodi’s MS environment by an RCB experiment with four replications (Exp. 6) whose management was identical to Exp. 4 and 5.

A first ANOVA that excluded parent line data and included the fixed factors line group and line within group and the random factor block aimed to compare the five groups of lines. A second ANOVA including also parent line data compared each line group to the mean value of parent lines by Dunnett’s multiple mean test.

## Figures and Tables

**Table 1:** Management, available water, air temperature in the last period of crop cycle and grain yield of pea experiments performed in a managed drought stress (MS) environment (Lodi, Italy) and two agricultural sites (Marchouch, Morocco; Alger, Algeria).

Exp.	Environment	Sowing Date ^1^	Harvest Date ^1^	Available Water (mm) ^2^	Last Month’s Mean Temperature (°C) ^3^	Mean Yield (t/ha)	Yield of Top-Yielding Line (t/ha)
Exp. 1	MS Lodi	Feb. 25, 2015	Jun. 3, 2015	120	19.3	0.32	0.75
Exp. 2	Marchouch	Nov. 28, 2015	May 26, 2016	59	18.1	0.36	0.91
Exp. 3	Alger	Dec. 8, 2015	May 18, 2016	327	20.3	1.38	3.33
Exp. 4, 5, 6	MS Lodi	Apr. 12, 2017	Jun. 24, 2017	115	23.4	0.42^4^	-

^1^ First sowing date and last harvest date, when spanning across various days. ^2^ Over the crop cycle; as irrigation under a rain-out shelter in Lodi, and rainfall in the other sites. ^3^ Average of mean daily temperature during the last month of crop cycle. ^4^ Mean of three experiments.

**Table 2:** Predicted efficiency (*E_r_*) relative to direct phenotypic selection (PS) for pea grain yield in the target environment of (i) indirect PS in a managed drought stress (MS) environment (Lodi, Italy) for two agricultural sites (Marchouch, Morocco; Alger, Algeria); (ii) genomic selection (GS) using a model trained on line yield data from the target environment (A) or on data averaged across the MS environment and Marchouch (B), for three environments.

Target Environment	*H_j_^2^*	*H_j’_^2^*	*r_g(j,j’)_*	*E_r_*, PS in MS ^a^	*r_Ab_* ^b^		*E_r_*, GS ^c^	
					A	B	A	B
Marchouch	0.475	0.870	0.408	0.550	0.240	0.260	0.633	0.685
Alger	0.522	0.870	0.015	0.021	0.184	0.031	0.441	0.074
MS Lodi	0.870	−	−	−	0.741	0.713	1.066	1.023

*H_j_^2^* and *H_j’_^2^*, broad sense heritability on a line mean basis for the target environment *j* and the selection environment *j’*, respectively, for PS; *r_g(j,j’)_*, genetic correlation for line yields across *j* and *j’* environments; *r_Ab_*, predictive ability of the top-performing of models constructed by Bayesian Lasso or Ridge Regression BLUP, considering models with five possible thresholds of genotype SNP missing data (10%, 20%, 30%, 40%, 50%) trained on joint data of three RIL populations (encompassing 288 lines overall). All values estimated for individual populations, reporting values averaged across populations. ^a^ Estimated as (*H_j_ H_j’_ r_g(jj’)_*)/*H_j_^2^*. ^b^ Top-predicting models are reported in Table 3 for A; they are BL models with missing data thresholds of 50% for Marchouch, 10% for Alger and 20% for MS Lodi, for B. ^c^ Estimated as (*i_j_’’ r_Ab_*)/(*i_j_ H_j_^2^*), where *i_j_’’* and *ij* are standardized selection differentials used for GS and PS, respectively; *i_j_’’* = 2.197 and *i_j_* = 1.755, upon assumption of same overall costs for GS and PS and 2.8 lower cost per evaluated line of GS relative to PS.

**Table 3:** Predictive ability of the top-performing of models constructed by Bayesian Lasso or Ridge Regression BLUP for grain yield breeding value of pea lines belonging to three connected RIL populations in a managed drought stress (MS) environment (Lodi, Italy) and two agricultural sites (Marchouch, Morocco; Alger, Algeria), with model training on all RIL populations pooled in one data set or on the single populations.

Trait	Bayesian Lasso		Ridge Regression BLUP	
	All	Single	All	Single
Yield, MS Lodi	0.741	0.708	0.707	0.693
Yield, Marchouch	0.240	0.214	0.240	0.217
Yield, Alger	0.181	0.156	0.184	0.160
Mean yield, MS Lodi and Marchouch ^1^	0.692	0.668	0.682	0.650

Averaged across results for three RIL populations encompassing 288 lines overall, considering models with five possible thresholds of genotype SNP missing data (10%, 20%, 30%, 40%, 50%). Fifty repetitions of 10-fold stratified cross-validations per analysis. ^1^ Using phenotypic data averaged across the two environments.

**Table 4:** Cross-population predictive ability of the top-performing of models constructed by Bayesian Lasso or Ridge Regression BLUP for breeding value of pea lines belonging to three connected RIL populations, for grain yield in the agricultural site of Alger (Algeria) and mean grain yield across a managed drought stress (MS) environment (Lodi, Italy) and the site of Marchouch (Morocco). Average predictions for one RIL based on model training on data of one or two other connected RIL populations.

Trait	Training Populations	
	One	Two
Yield, Alger	0.099	0.151
Mean yield, MS Lodi, and Marchouch ^1^	0.397	0.630

Averaged across results for three RIL populations encompassing 288 lines overall, considering models with five possible thresholds of genotype SNP missing data (10%, 20%, 30%, 40%, 50%). ^1^ Using phenotypic data averaged across the two environments.

**Table 5:** Grain yield, aerial biomass, and onset of flowering under managed drought stress (MS) of pea line groups issued by genomic selection (GS) or phenotypic selection (PS) for grain yield under severe terminal drought or marker-assisted selection (MAS) for intrinsic drought tolerance.

Line Group	Total no. of Lines	Yield (t/ha Dry Weight)		Aerial Biomass (t/ha Dry Weight)	Onset of Flowering (dd from April 1)
		Value	Difference to Parent Line Group		
**Experiment 4 ^1^**
PS in MS Lodi	9	0.749 **	0.495	3.264 **	26.2 **
GS, RIL population-specific model	9	0.655 **	0.401	3.299 **	27.4 **
PS across MS Lodi and Marchouch	9	0.653 **	0.399	3.216 *	27.4 **
GS, model trained on all populations	9	0.642 **	0.388	3.015	26.2 **
PS in Marchouch	9	0.540 **	0.286	3.094	28.5 **
Parent lines	3	0.254	-	2.819	30.8
LSD (*p* < 0.05)		0.104		0.195	0.5
**Experiment 5 ^2^**
GS, top-performing lines	6	0.353 *	0.128	2.786	31.2
GS, mid-performing lines	6	0.134	−0.091	2.747	35.5 **
GS, bottom-performing lines	6	0.121	−0.104	2.581	35.3 **
Parent lines	3	0.225	-	2.686	32.7
LSD (*p* < 0.05)		0.068		0.253	1.5
**Experiment 6 ^3^**
GS, top-performing lines	9	0.638 **	0.286	3.375 **	28.8
MAS, top-performing lines	9	0.595 **	0.243	3.031 **	28.3
GS/MAS mid-performing lines	6	0.462	0.110	3.059 **	28.6
GS, bottom-performing lines	9	0.290	−0.062	2.649	29.3
MAS, bottom-performing lines	9	0.208	−0.144	2.597	30
Parent lines	2	0.352	-	2.506	28.6
LSD (*p* < 0.05)		0.114		0.297	1.0

GS modelling based on data of independent lines evaluated in a MS experiment in Lodi and a field experiment in Marchouch (Exp. 1 and 2 in Table 1, respectively). LSD relates to line group mean comparison, excluding parent lines. Line group means followed by * and ** differ at *p* < 0.05 and *p* < 0.01, respectively, from the parent line mean according to Dunnett’s test. ^1^ GS model trained on 205 lines from three RIL populations or on the single populations. GS and PS selection: three lines out of 30, for each of three RIL populations. GS and PS data averaged across populations. ^2^ GS model trained on 295 lines from three RIL populations. Each GS-based line group: two lines out of 30, for each of three connected crosses. GS data averaged across connected crosses. ^3^ GS model trained on 198 lines from two RIL populations. GS and MAS selection: applied to 24 lines previously selected for similar phenology out of 97 lines from another RIL population, selecting three lines for top- and bottom-performing groups, and two lines for the mid-performing group.

## Supplementary Materials

**Supplementary Materials** can be found at https://www.mdpi.com/1422-0067/21/7/2414/s1. **Table S1:** AMMI analysis for grain yield of 288 lines belonging to three connected RIL populations, three parent cultivars and one recent control cultivar grown in a managed drought stress environment of Lodi (Italy) and two agricultural environments of Marchouch (Morocco) and Alger (Algeria). **Table S2:** Cross-environment predictive ability (*r_Ab_*) and predictive accuracy (*r_Ac_*) of the top-performing of models constructed by Bayesian Lasso (BL) or Ridge Regression BLUP (rrBLUP) for grain yield breeding value of pea lines belonging to three connected RIL populations in a managed drought stress (MS) environment (Lodi, Italy) and two agricultural sites (Marchouch, Morocco; Alger, Algeria). **Table S3:** ANOVA *F* test results for grain yield, aerial biomass, and onset of flowering under managed drought stress (MS) of pea line groups belonging to different RIL populations or crosses. **Table S4:** Markers associated with intrinsic drought tolerance (as grain yield deviation from the value expected according to onset of flowering) and their location in five genomic areas of Tayeh et al.’s [73] consensus map and on Kreplak et al.’s [75] pea reference genome (for which we report also the name of the gene coding region). Donor genotype: A = Attika; I = Isard; K = Kaspa. See Supplementary Table 1 in Annicchiarico et al. [30] for SNP marker sequence. **Figure S1:** Predictive ability of two genomic selection models (Bayesian Lasso, BL, and Ridge Regression BLUP, rrBLUP) and five thresholds of genotype SNP missing data (10%, 20%, 30%, 40%, 50%), for grain yield of pea lines belonging to three connected RIL populations in a managed drought stress (MS) environment of Lodi (Italy) and the agricultural environments of Marchouch (Morocco) and Alger (Algeria) and for mean grain yield across the MS environment and Marchouch. Model training on data of three RIL populations (encompassing 288 lines overall), averaging validation results for individual populations based on 50 repetitions of 10-fold stratified cross-validations per individual analysis. **Data repository S1:** Phenotypic and genotypic data. Grain yield in three environments, and SNP marker data for five thresholds of genotype SNP missing data (10%, 20%, 30%, 40%, 50%), for 288 pea lines belonging to three connected RIL populations.

## Abbreviations

rrBLUP	Ridge Regression BLUP (best linear unbiased prediction)
ANOVA	Analysis of variance
AMMI	Additive Main effects and Multiplicative Interaction
GBS	Genotyping-by-sequencing
MAS	Marker-assisted selection
RIL	Recombinant inbred line
SNP	Single nucleotide polymorphism
BL	Bayesian Lasso
GE	Genotype × environment
GS	Genomic selection
MS	Managed stress
PC	Principal component
PS	Phenotypic selection
