# Supplementary material for: Development and Proof-of-Concept Application of Genome-Enabled Selection for Pea Grain Yield under Severe Terminal Drought
Source: Int J Mol Sci. 2020 Mar 31;21(7):2414. doi: 10.3390/ijms21072414 (PMC7177262; doi:10.3390/ijms21072414)
Supplement: Supplementary file 1 [file ijms-21-02414-s001.zip › ijms-732093-Supplementary Materials-to conversion/ijms-732093-Supplementary tables and figures.pdf]

International Journal of *Molecular Sciences*

Supplementary Materials:

Development and Proof-Of-Concept Application of Genome-Enabled Selection for Pea Grain Yield under Severe Terminal Drought

Paolo Annicchiarico [1,*], Nelson Nazzicari [1], Meriem Laouar [2], Imane Thami-Alami [3], Massimo Romani [1] and Luciano Pecetti [1]

1 Council for Agricultural Research and Economics (CREA), Research Centre for Animal Production and Aquaculture, viale Piacenza 29, 26900 Lodi, Italy; nelson.nazzicari@crea.gov.it (N.N.); mas.romani@libero.it (M.R.); luciano.pecetti@crea.gov.it (L.P.)
2 Ecole Nationale Supérieure Agronomique (ENSA), Laboratoire d'Amélioration Intégrative des Productions Végétales (C2711100), Rue Hassen Badi, El Harrach, DZ16200 Alger, Algeria; laouar_m@yahoo.fr
3 Institut National de la Recherche Agronomique (INRA), Centre Régional de Rabat, Av. de la Victoire, BP 415 Rabat, Morocco; thamialami_ma@yahoo.fr
* Correspondence: paolo.annicchiarico@crea.gov.it

Data repository S1: Phenotypic and genotypic data. Grain yield in three environments, and SNP marker data for five thresholds of genotype SNP missing data (10%, 20%, 30%, 40%, 50%), for 288 pea lines belonging to three connected RIL populations.

Table S1. AMMI analysis for grain yield of 288 lines belonging to three connected RIL populations, three parent cultivars and one recent control cultivar grown in a managed drought stress environment of Lodi (Italy) and two agricultural environments of Marchouch (Morocco) and Alger (Algeria).

Source of Variation	Degrees of Freedom	Mean Square
Genotype	291	0.370 ***
Environment	2	273.091 ***
Block (Environment)	7	3.102
Genotype × Environment interaction	582	0.430 ***
- PC 1	292	0.798 ***
- Residual	290	0.059 NS
Pooled experimental error	2037	0.188

***: significant at $P < 0.001$; NS: not significant ($P > 0.05$).

Table S2. Cross-environment predictive ability (r_{Ab}) and predictive accuracy (r_{Ac}) of the top-performing of models constructed by Bayesian Lasso (BL) or Ridge Regression BLUP (rrBLUP) for grain yield breeding value of pea lines belonging to three connected RIL populations in a managed drought stress (MS) environment (Lodi, Italy) and two agricultural sites (Marchouch, Morocco; Alger, Algeria).

Predicted Environment	Training Environment	r_{Ab}	r_{Ac}	Model (Missing Rate)
MS Lodi	Marchouch	0.430	0.461	rrBLUP (40%)
MS Lodi	Alger	0.049	0.053	rrBLUP (20%)
Marchouch	MS Lodi	0.244	0.354	BL (40%)
Marchouch	Alger	0.077	0.112	BL (10%)
Alger	MS Lodi	0.034	0.047	rrBLUP (20%)
Alger	Marchouch	0.037	0.051	BL (10%)

Values for the top-performing model averaged across results for three RIL populations, considering models trained on joint data of the RIL populations (encompassing 288 lines overall) with five possible thresholds of genotype SNP missing data (10%, 20%, 30%, 40%, 50%). Fifty repetitions of 10-fold stratified cross-validations per analysis.

Table S3. ANOVA *F* test results for grain yield, aerial biomass and onset of flowering under managed drought stress (MS) of pea line groups belonging to different RIL populations or crosses.

Source of Variation[a]	Degrees of Freedom	Grain Yield	Aerial Biomass	Onset of Flowering
**Experiment 4**				
Line Group	4	**	*	**
RIL population	2	**	NS	**
Line within Group and RIL population	30	**	**	**
Line Group × RIL population interaction	8	NS	NS	**
**Experiment 5**				
Line Group	2	**	NS	**
Cross	2	**	**	**
Line within Group and Cross	9	**	*	**
Line Group × Cross interaction	4	*	**	NS
**Experiment 6**				
Line Group	4	**	**	*
Line within Group	9	**	**	*

*, **: significant at $P < 0.05$ and $P < 0.01$, respectively. [a] The ANOVAs included the line groups described for each experiment in Table 4, except the parent line group.

Table S4. Markers associated with intrinsic drought tolerance (as grain yield deviation from the value expected according to onset of flowering) and their location in five genomic areas of Tayeh et al.'s [73] consensus map and on Kreplak et al.'s [75] pea reference genome (for which we report also the name of the gene coding region). Donor genotype: A = Attika; I = Isard; K = Kaspa. See Supplementary Table 1 in Annicchiarico et al. [30] for SNP marker sequence.

Marker	−log(p)	Donor	Tayeh Map		Kreplak Map		
			LG	cM	Chromosome	Position	Gene coding region
TP78343	2.98	A	LG5	110.6	chr3LG5	432274508	Psat3g204440 (Mitochondrial carrier protein)
TP13485	2.74	A	LG5	108.4	chr3LG5	429763510	Psat3g203440 (Chromosome condensation regulator RCC1 signature)
TP94476	2.47	I	LG1	35.8	chr2LG1	96919562	Psat0s1135g0040 (Transcription initiation factor TFIID signature)
TP6268	2.44	A	LG3	57.2	chr5LG3	193175134	Psat5g108880 (OPT oligopeptide transporter protein)
TP63677	2.31	K	LG7	32.2	chr7LG7	100049395	Psat7g060200 (Protein kinase domain)
TP51372	2.31	K	LG7	32.8	chr7LG7	102333629	Psat7g061720 (NAD binding domain of 6-phosphogluconate dehydrogenase)
TP6885	2.25	K	LG7	76.7	chr7LG7	352130796	Psat7g188520 (Phosphotyrosyl phosphate activator (PTPA) protein)

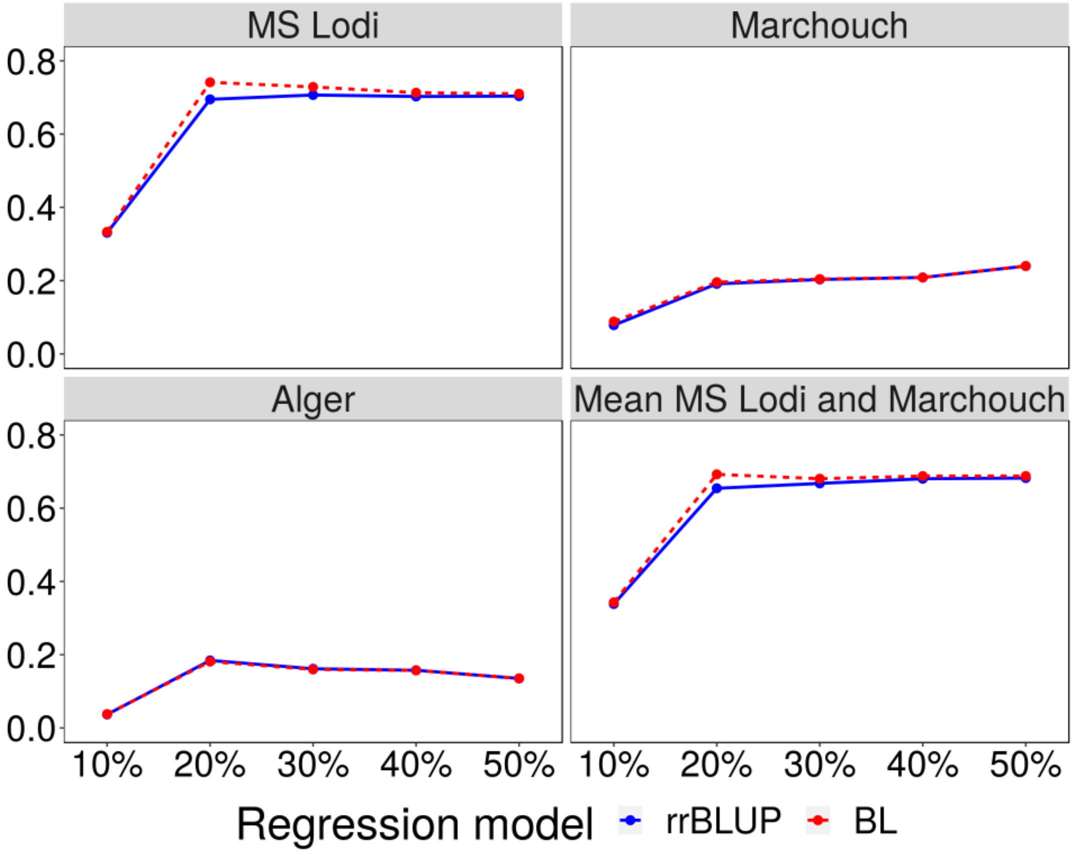

Figure S1. Predictive ability of two genomic selection models (Bayesian Lasso, BL, and Ridge Regression BLUP, rrBLUP) and five thresholds of genotype SNP missing data (10%, 20%, 30%, 40%, 50%), for grain yield of pea lines belonging to three connected RIL populations in a managed drought stress (MS) environment of Lodi (Italy) and the agricultural environments of Marchouch (Morocco) and Alger (Algeria) and for mean grain yield across the MS environment and Marchouch. Model training on data of three RIL populations (encompassing 288 lines overall), averaging validation results for individual populations based on 50 repetitions of 10-fold stratified cross-validations per individual analysis.
